# Supplementary material for: Content-rich biological network constructed by mining PubMed abstracts
Source: BMC Bioinformatics. 2004 Oct 8;5:147. doi: 10.1186/1471-2105-5-147 (PMC528731; doi:10.1186/1471-2105-5-147)
Supplement: Additional File 2 — The original results of the above study (non-essential files are deleted to keep the file size under the limit set by BMC bioinformatics). [file 1471-2105-5-147-S2.bz2 › chilibotAdditionalFile2/dip05/201ID8652557E690/html/ERCC6_ERCC5.html]

 


 **ERCC6** and **ERCC5** 
  
Found 21 abstracts in PubMed, retrieved 05.  
 

 What does Google say? 
 PDF only 
| .edu only 

---

**Interactive relationship** (e.g. stimulation, inhibition, etc)

**Neutral relationship**- Here, we provide evidence that CSB  [ **ERCC6** ]  is found not only in the nucleoplasm but also in the nucleolus within a complex CSB  [ **ERCC6** ]  IP 150 that contains RNA pol I, TFIIH, and XPG  [ **ERCC5** ]  and promotes efficient rRNA synthesis.  Ref: 12419226 Mol Cell, 2002

**Non-interactive relationship** (e.g. studied together, co-existance, homology, etc.)

- In multivariate logistic regression analysis, low expression of ERCC1, XPB ERCC3, XPG  [ **ERCC5** ]   **ERCC5** , and CSB  [ **ERCC6** ]   **ERCC6**  was associated with a statistically significant increased risk for SCCHN.  Ref: 11900225 Cancer, 2002
- CONCLUSIONS Reduced expression of ERCC1, XPB ERCC3, XPG  [ **ERCC5** ]   **ERCC5** , and CSB  [ **ERCC6** ]   **ERCC6**  is associated with a more than two fold increased risk of SCCHN.  Ref: 11900225 Cancer, 2002
- In contrast to cells bearing mutations in other components of the nucleotide excision repair apparatus XPB, XPD, XPG  [ **ERCC5** ]  and CSB  [ **ERCC6** ] , cells defective for the ERCC1 XPF structure specific nuclease are highly sensitive to cisplatin.  Ref: 12202770 Nucleic Acids Res, 2002
- RESULTS The levels of ERCC1, XPB ERCC3, XPG  [ **ERCC5** ]   **ERCC5** , and CSB  [ **ERCC6** ]   **ERCC6**  transcripts were lower in cases than in controls.  Ref: 11900225 Cancer, 2002
